# Transglutaminase-crosslinked extracellular matrix in cirrhotic liver tissues provides pro-fibrotic mechano-microenvironment for cells

**DOI:** 10.1093/lifemedi/lnad030

**Published:** 2023-08-23

**Authors:** Cheng Lyu, Wenyu Kong, Yan Zhang, Zhiqiang Liu, Kaini Liang, Yanan Du

**Affiliations:** Department of Biomedical Engineering, School of Medicine, Tsinghua-Peking Center for Life Sciences, Tsinghua University, Beijing 100084, China; Department of Biomedical Engineering, School of Medicine, Tsinghua-Peking Center for Life Sciences, Tsinghua University, Beijing 100084, China; Department of Biomedical Engineering, School of Medicine, Tsinghua-Peking Center for Life Sciences, Tsinghua University, Beijing 100084, China; Department of Biomedical Engineering, School of Medicine, Tsinghua-Peking Center for Life Sciences, Tsinghua University, Beijing 100084, China; Department of Biomedical Engineering, School of Medicine, Tsinghua-Peking Center for Life Sciences, Tsinghua University, Beijing 100084, China; Department of Biomedical Engineering, School of Medicine, Tsinghua-Peking Center for Life Sciences, Tsinghua University, Beijing 100084, China

**Dear Editor,**

Liver cirrhosis is featured by the highly crosslinked extracellular matrix (ECM), mainly consisting of collagen scars. Increased crosslinking degree of collagen scar is associated with its resistance to proteolytic degradation and more difficulties in fibrosis recovery [1]. The only reliable clinical treatment for liver cirrhosis is still the liver transplantation. Crosslinked scars can damage the liver tissues and generate abnormal mechanical cues that modulate the pro-fibrotic phenotypes of cells [2]. Multiple crosslinking reactions involve in the formation of collagen scars, and the most widely reported one is the lysyl oxidase (LOX)-mediated crosslinking. Disappointingly, the failure of LOX-targeting therapeutics in clinical trials calls for further investigation of crosslinked collagen scar in liver cirrhosis [3]. In addition to LOX, tissue transglutaminase (TGM)-mediated crosslinking has also been reportedly responsible for the ECM crosslinking in fibrotic liver tissues. TGM enzymatically catalyzes the formation of γ-Glutamyl-ε-Lysine (γ-GLU-ε-LYS) isopeptide crosslinks, resulting in the increased stability, irreversibility, and resistance to remodeling of collagen scar [2]. However, there is no on-market anti-TGM therapeutic for the treatment of liver cirrhosis, and it is still unclear how the TGM crosslinking modulates the mechano-microenvironment in the fibrotic liver tissue. Therefore, thorough characterization and reconstruction of TGM-crosslinked ECM in cirrhotic liver tissues is greatly in need and is promising for exploiting the downstream cellular responses during pathogenesis.

Remarkable collagen scar tissues and an extremely high expression of TGM were found in human cirrhotic liver tissues, which was not observed in healthy tissues (Fig. 1A). In a liver fibrosis mouse model, dramatic increases of collagen scar tissue, TGM, and TGM-specific crosslinks, γ-GLU-ε-LYS, could be observed with prolonged fibrosis, indicating that TGM could potentially crosslink the collagen scar through generating specific crosslinks in fibrotic liver tissues (Fig. 1B–E).

**Figure 1.**
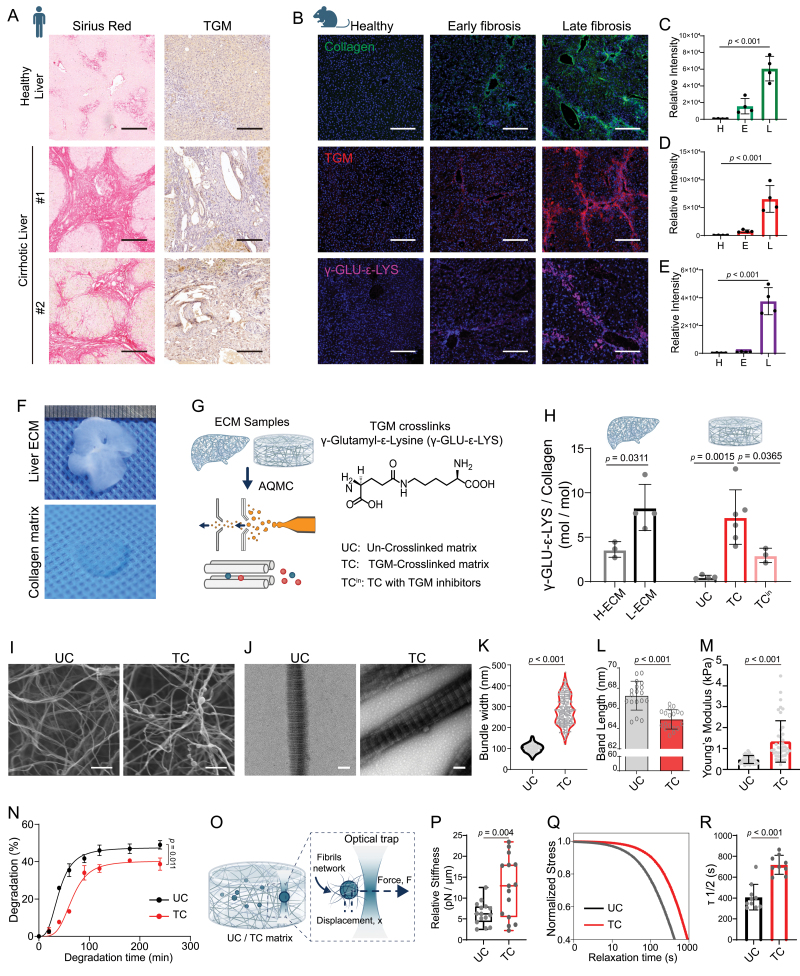
**Characterization and *in vitro* reconstruction of TGM-crosslinked collagen matrix in liver cirrhosis.**(A) Representative Sirius Red and TGM histological staining of healthy and cirrhotic human liver samples. Scale bar, 200 μm. (B) Representative immunofluorescent staining of collagen, TGM, and γ-GLU-ε-LYS in liver tissue samples obtained from mice with different liver fibrosis stages. Scale bar, 200 μm. (C–E) Statistical analysis of relative intensity of collagen (C), TGM (D), and γ-GLU-ε-LYS (E) staining as shown in (B) (*n* ≥ 3, randomly selected fields per condition). (F) Representative bright field images of mouse liver decellularized ECM and reconstructed collagen matrix. (G) Schematic of characterizing the TGM crosslinking degree of liver ECM and collagen matrix by using AQMC method. (H) Quantitative analysis of TGM crosslinking degree of liver ECM and collagen matrix. UC, Un-crosslinked collagen matrix; TC, TGM-crosslinked collagen matrix; TC^in^, TC with additional inhibitors to inhibit TGM crosslinking (*n* ≥ 3, pieces of different ECM or collagen matrix samples). (I) Representative SEM images of UC and TC collagen matrix. Scale bar, 1 μm. (J) Representative TEM images of UC and TC collagen fibrils. Scale bar, 100 nm. (K) Statistical analysis of width of tangled fibril bundles (*n* ≥ 30, randomly selected fibrils). (L) Statistical analysis of Band length of collagen fibrils as shown in TEM images (*n* ≥ 30, randomly selected samples). (M) Young’s modulus of UC and TC matrix detected by AFM indentation test (*n* ≥ 30, randomly selected points of measurement). (N) Collagenase-mediated degradation rate of UC and TC matrix (*n* = 3, independent samples per condition). (O) Schematic of characterizing stress relaxation rate of UC and TC matrix by using optical tweezers. (P) Matrix stiffness of the collagen matrix (*n* = 15, points of detection per condition). (Q) Representative stress relaxation curve of the UC and TC collagen matrix. (R) Statistical analysis of timescale at which the stress is relaxed to half its original value, *τ*1/2, from stress relaxation tests in (Q) (*n* ≥ 9, points of detection per condition). Statistical significance was determined using a two-tailed unpaired Student’s *t*-test for the comparison of two groups or ANOVA a one-way ANOVA with Turkey test for comparison between multiple groups. Data are presented as the mean with standard deviation.

In order to reconstruct TGM-crosslinked matrix *in vitro* models, we used a previously developed method, Absolute Quantification of Matrix-specific Crosslinking (AQMC) [4], to precisely quantify the TGM crosslinking degree of liver ECM in fibrotic mice. We reconstructed the TGM-crosslinked collagen matrix *in vitro* with the instructions of *in vivo* parameters (Fig. 1F–H). Tissue decellularization was performed to obtain the liver ECM (Fig. 1F). We next quantified the TGM crosslinks (e.g., γ-GLU-ε-LYS) by using Liquid Chromatography with tandem mass spectrometry (LC-MS/MS) based strategy (Fig. 1G). The degree of TGM crosslinking was significantly higher in late-fibrotic ECM compared to healthy ECM, demonstrating that TGM crosslinking contributed to the formation of crosslinked scar tissue. By inducing TGM crosslinking reactions in collagen matrix *in vitro*, we successfully prepared TGM-crosslinked collagen matrix (TC) with increased crosslinking degree compared to previous study [4], and the crosslinking degree of TGM-crosslinked collagen matrix was comparable to that of late-fibrotic liver ECM. The un-crosslinked collagen matrix (UC) showed a very low level of TGM crosslinking degree, which was used to model the healthy liver ECM. The additional treatment of TGM-specific inhibitors led to a decrease in the crosslinking degree of TGM-crosslinked collagen matrix.

The collagen fibrils in the un-crosslinked collagen matrix were sparsely distributed. Whereas a number of nodular structures inter-connecting the adjacent fibrils could be observed in TGM-crosslinked matrix (Fig. 1I). TGM-crosslinked fibrils tended to align together with increased dimension (Fig. 1J and 1K). Additionally, a decrease in the length of the D-period in the TGM-crosslinked fibrils compared to un-crosslinked fibrils was observed (Fig. 1L).

The TGM-crosslinked matrix exhibited a higher elastic modulus and greater resistance to collagenase-mediated degradation than the un-crosslinked matrix (Fig. 1M and 1N). Furthermore, the optical tweezer assay was used to probe the viscoelasticity of collagen matrix, which revealed that the TGM-crosslinked collagen matrix showed a higher stiffness and a significantly longer stress relaxation time than the un-crosslinked matrix (Fig. 1O–R).

Taken together, the reconstructed TGM-crosslinked collagen matrix reproduced crosslinked ECM mediated by TGM in fibrotic liver tissues, with similar TGM crosslinking degree, revealing the abnormal changes in matrix biomechanics and the pro-disease mechanical cues during fibrosis progression.

Since the liver sinusoidal endothelial cells (LSECs) and macrophages play regulatory roles in liver pathophysiology, and their phenotypes and functions are tunable in response to the mechano-microenvironment [5], we next sought to investigate the regulatory effects of TGM-crosslinked matrices on LSECs and macrophages by using the *in-vivo*-mimicking collagen matrix models.

LSECs have fenestrated structures and loosely contact the ECM membrane in healthy liver tissues, which enables the highly active protein transport across the endothelial layers. We found that LSECs grown on the TGM-crosslinked matrix showed increased expression of focal adhesion molecules compared to the un-crosslinked group (Fig. 2A–B and 2D). This indicated the elevated cell–matrix interactions, one of the pathological features of LSECs in fibrotic tissues [5, 6]. In addition, the loss of endothelial adherent junction protein was reported in LSECs with fibrotic and cirrhotic phenotypes [6]. We observed that integrin β2, a molecule highly involved in cell–cell adhesion, was highly expressed in LSECs grown on un-crosslinked matrix, especially in the cell–cell contacting regions (Fig. 2C and 2E). Whereas no integrin β2-mediated tight junctions between adjacent LSECs grown on TGM-crosslinked matrix could be observed, indicating decreased cell–cell junctions mediated by TGM-crosslinked matrix. Yes-associated protein (YAP) is a mechano-sensitive epigenetic regulator related to the dysfunction of cirrhosis [7, 8]. LSECs grown on TGM-crosslinked matrix showed an obvious increase in nuclear YAP expression (Fig. 2F and 2G). LSECs grown on TGM-crosslinked matrix showed higher mRNA expression of genes related to cell–ECM adhesion (i.e., *DDR2*) and fibrotic response (i.e., *PDGFA* and *COL3A1*) compared to un-crosslinked group (Fig. 2H).

**Figure 2.**
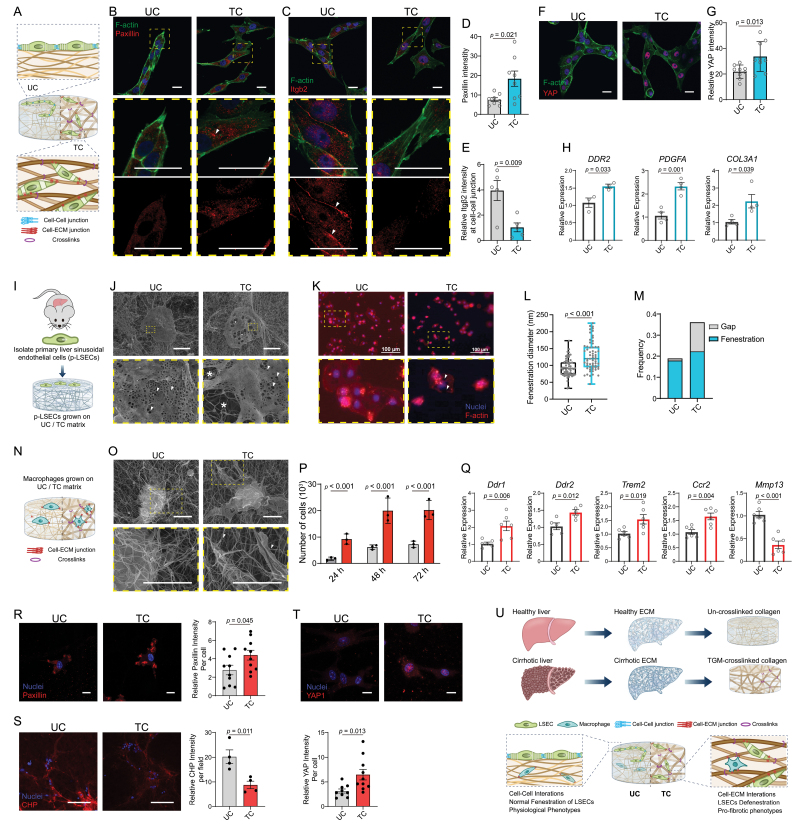
**TGM-crosslinked collagen matrix led to abnormally upregulated cell–matrix interactions in LSECs and macrophages and pro-fibrotic cellular responses.**(A) Schematic of LSECs grown on UC and TC matrix. (B) Representative images of paxillin expression of LSECs grown on UC and TC matrix. Tips cells are marked by asterisk. Top panels, overall view; Middle panels, detailed view showing strong paxillin expression at actin-rich cell adhesion regions as indicated by arrows; bottom panels, separated paxillin channels of middle panels. Scale bars, 20 μm. (C) Representative images of integrin β2 expression of LSECs grown on UC and TC matrix. Top panels, overall view; Middle panels, detailed view; bottom panels, separated integrin β2 channels of middle panels, showing strong integrin β2 expression at cell–cell junction regions as indicated by arrows. Scale bars, 20 μm. (D) Relative paxillin intensity per cell that co-localized with actin signals as shown in (B) (*n* = 8, number of cells analyzed per condition). Scale bars, 20 μm. (E) Relative integrin β2 intensity at cell–cell junction regions as shown in (C) (*n* = 5, number of cells analyzed per condition). (F) Representative images of YAP expression in LSECs grown on UC and TC matrix. Scale bars, 20 μm. (G) Relative YAP intensity per cell as shown in (F) (*n* = 9, number of cells analyzed per condition). (H) Relative mRNA expression of *DDR2*, *PDGFA*, *COL3A1* in LSECs grown on UC and TC collagen matrix (*n* ≥ 3, biologically independent samples). (I) Schematic of isolating and culturing mouse primary liver sinusoidal endothelial cells (p-LSECs) on reconstructed collagen matrix. (J) Representative SEM images of p-LSECs grown on UC and TC collagen matrix. The fenestration structures are indicated by arrows. The abnormal gap structures are marked by asterisks. Scale bars, 10 μm. (K) Representative F-actin staining of p-LSECs grown on UC and TC collagen matrix. The abnormal gap structures are marked by arrows. Scale bars, 100 μm. (L) Statistical analysis of the diameter of fenestration structures (*n* ≥ 61). (M) Fraction of fenestration structures and Gap structures in p-LSECs grown on UC and TC collagen matrix. (N) Schematic of macrophages grown on UC and TC collagen matrix. (O) Representative SEM images of macrophages grown on UC and TC matrix. The arrows indicate the strong cell protrusion structures binding the collagen fibrils. Scale bars, 5 μm. (P) Macrophages adhesion test showing number of cells adhering to UC and TC matrix (*n* = 3, biologically independent samples). (Q) Relative mRNA expression of *Ddr1*, *Ddr2*, *Trem2*, *Ccr2*, and *Mmp13* in macrophages grown on UC and TC collagen matrix (*n* ≥ 5, biologically independent samples). (R) Representative images and statistical analysis of paxillin expression of macrophages grown on UC and TC matrix (*n* = 10). Scale bars, 10 μm. (S) Representative images and statistical analysis of CHP intensity (*n* = 4). Scale bars, 200 μm. (T) Representative images and statistical analysis of YAP expression of macrophages grown on UC and TC matrix (*n* = 10). Scale bars, 10 μm. (U) Graphical summary. Statistical significance was determined using a two-tailed unpaired Student’s *t*-test. Data are presented as the mean with standard deviation.

We then investigated how the fenestration structure of LSECs, a critical feature of liver sinusoids responsible for mass transport, was regulated by the TGM-crosslinked matrix. We isolated and cultured mouse primary liver sinusoidal endothelial cells (p-LSECs) on reconstructed collagen matrix (Fig. 2I and Fig. S1). The p-LSECs grown on un-crosslinked matrix have uniformly distributed small fenestrae of 100–200 nm in size. However, many small fenestrae merged together, forming large gap structures of ~1 μm in size, which was observed in p-LSECs grown on TGM-crosslinked matrix (Fig. 2J and 2K). The loss of fenestration structure and the dramatic increase of gaps were observed in TGM-crosslinked group (Fig. 2L and 2M), which was reportedly related to cellular response of LSECs to chronic liver disease.

These results showed that the aberrant mechano-microenvironment in TGM-crosslinked matrix resulted in abnormally downregulated cell–cell adhesions and upregulated cell–ECM adhesions in LSECs. These mechano-signals led to the increased expression of YAP and upregulated fibrosis-related gene expression. The pathological loss of fenestration was also validated by p-LSECs primed by TGM-crosslinked matrix.

We then moved on to exploit how the TGM-crosslinked matrix modulated the phenotypes of macrophages which showed active interactions with ECM in liver tissue and contributed to disease progression through modulating immune response (Fig. 2N). Macrophages grown on TGM-crosslinked matrix could form more protrusion-like structures binding to adjunct collagen fibrils, indicating strong cell–ECM junctions (Fig. 2O). Much more suspending macrophages could adhere to TGM-crosslinked matrix than un-crosslinked group in a period of time (Fig. 2P). Macrophages grown on TGM-crosslinked matrix showed higher levels of expression of genes related to cell–ECM interactions (i.e., *Ddr1* and *Ddr2*) and markers related to cirrhotic tissues (i.e., *Trem2* and *Ccr2*) [9]. The gene expression related to fibrosis reversal (i.e., *Mmp13*) was downregulated in macrophages grown on TGM-crosslinked matrix (Fig. 2Q).

Consistently, macrophages grown on TGM-crosslinked matrix showed a higher level of ECM adhesion molecules compared to the un-crosslinked group (Fig. 2R). Collagen Hybridizing Peptide assay, which was used to visualize the collagen matrix being degrading by the adhered cells, showed that macrophage-mediated collagen degradation was exceptionally decreased in that of TGM-crosslinked matrix (Fig. 2S). An increase of YAP expression in macrophages grown in TGM-crosslinked matrix was observed compared to the un-crosslinked group (Fig. 2T). The abnormal YAP expression in macrophages could also be caused by decreased stress relaxation of matrix, and was related to dysregulated immune responses in disease progression [10]. These results indicated that macrophages grown on TGM-crosslinked matrix showed increased cell–ECM interactions, YAP expression, and decreased ECM-degradation abilities compared to that grown on un-crosslinked matrix.

In summary, we validated the high level of TGM crosslinking characteristics of ECM in cirrhotic liver tissues, and reconstructed TGM-crosslinked collagen matrix *in vitro*, which faithfully reproduced the TGM crosslinking degree of fibrotic liver ECM. TGM crosslinking increased the stiffness and decreased the stress relaxation rate of the matrix, which constitutes an abnormal mechano-microenvironment for cells. By using the *in-vivo*-mimicking models, we revealed that LSECs and macrophages grown on TGM-crosslinked matrix showed increased cell–ECM junctions, YAP expression, and fibrosis-related phenotypes. Our results provide new insights for mechanism research and clinical applications through reconstructing ECM-based disease models.

## Research limitations

We only focused on TGM crosslinking in this study, which could not totally reproduce the crosslinked scar caused by multiple types of crosslinking mechanisms in cirrhotic liver tissues. Type I Collagen is the only type of composition of ECM used for the *in vitro* study, whereas other ECM components in liver tissue, such as elastin and laminin, were not considered. The spatial cues of cell–ECM interactions that mimic the liver sinusoidal microenvironment are not entirely reproduced in the current *in vitr*o models.

## Supplementary Material

lnad030_suppl_Supplementary_Material

## References

[1] Pellicoro A, Ramachandran P, Iredale JP, et al. Liver fibrosis and repair: immune regulation of wound healing in a solid organ. Nat Rev Immunol 2014;14:181–94. 24566915 10.1038/nri3623

[2] Kong W, Lyu C, Liao H, et al. Collagen crosslinking: effect on structure, mechanics and fibrosis progression. Biomed Mater 2021;16:6. 10.1088/1748-605X/ac2b79 34587604

[3] Lampi MC, Reinhart-King CA. Targeting extracellular matrix stiffness to attenuate disease: From molecular mechanisms to clinical trials. Sci Transl Med 2018;10:eaao0475. 29298864 10.1126/scitranslmed.aao0475

[4] Lyu C, Kong W, Liu Z, et al. Advanced glycation end-products as mediators of the aberrant crosslinking of extracellular matrix in scarred liver tissue. Nat Biomed Eng 2023. doi:10.1038/s41551-023-01019-z. 10.1038/s41551-023-01019-z 37037967

[5] Liu LW, You ZF, Yu HS, et al. Mechanotransduction-modulated fibrotic microniches reveal the contribution of angiogenesis in liver fibrosis. Nat Mater 2017;16:1252. 29170554 10.1038/nmat5024

[6] Su TT, Yang YL, Lai SC, et al. Single-cell transcriptomics reveals zone-specific alterations of liver sinusoidal endothelial cells in cirrhosis. Cell Mol Gastroenter 2021;11:1139–61. 10.1016/j.jcmgh.2020.12.007 PMC7903131 33340713

[7] Herrera J, Henke CA, Bitterman PB. Extracellular matrix as a driver of progressive fibrosis. J Clin Invest 2018;128:45–53. 29293088 10.1172/JCI93557 PMC5749528

[8] Choi HJ, Kwon YG. Roles of YAP in mediating endothelial cell junctional stability and vascular remodeling. Bmb Rep 2015;48:429–30. 26169195 10.5483/BMBRep.2015.48.8.146 PMC4576949

[9] Ramachandran P, Dobie R, Wilson-Kanamori JR, et al. Resolving the fibrotic niche of human liver cirrhosis at single-cell level. Nature 2019;575:512–8. 31597160 10.1038/s41586-019-1631-3 PMC6876711

[10] Meli VS, Atcha H, Veerasubramanian PK, et al. YAP-mediated mechanotransduction tunes the macrophage inflammatory response. Sci Adv 2020;6:eabb8471. 33277245 10.1126/sciadv.abb8471 PMC7717914

