# Supplementary material for: Transglutaminase-crosslinked extracellular matrix in cirrhotic liver tissues provides pro-fibrotic mechano-microenvironment for cells
Source: Life Med. 2023 Aug 23;2(6):lnad030. doi: 10.1093/lifemedi/lnad030 (PMC11748972; doi:10.1093/lifemedi/lnad030)
Supplement: lnad030_suppl_Supplementary_Material [file lnad030_suppl_Supplementary_Material.pdf]

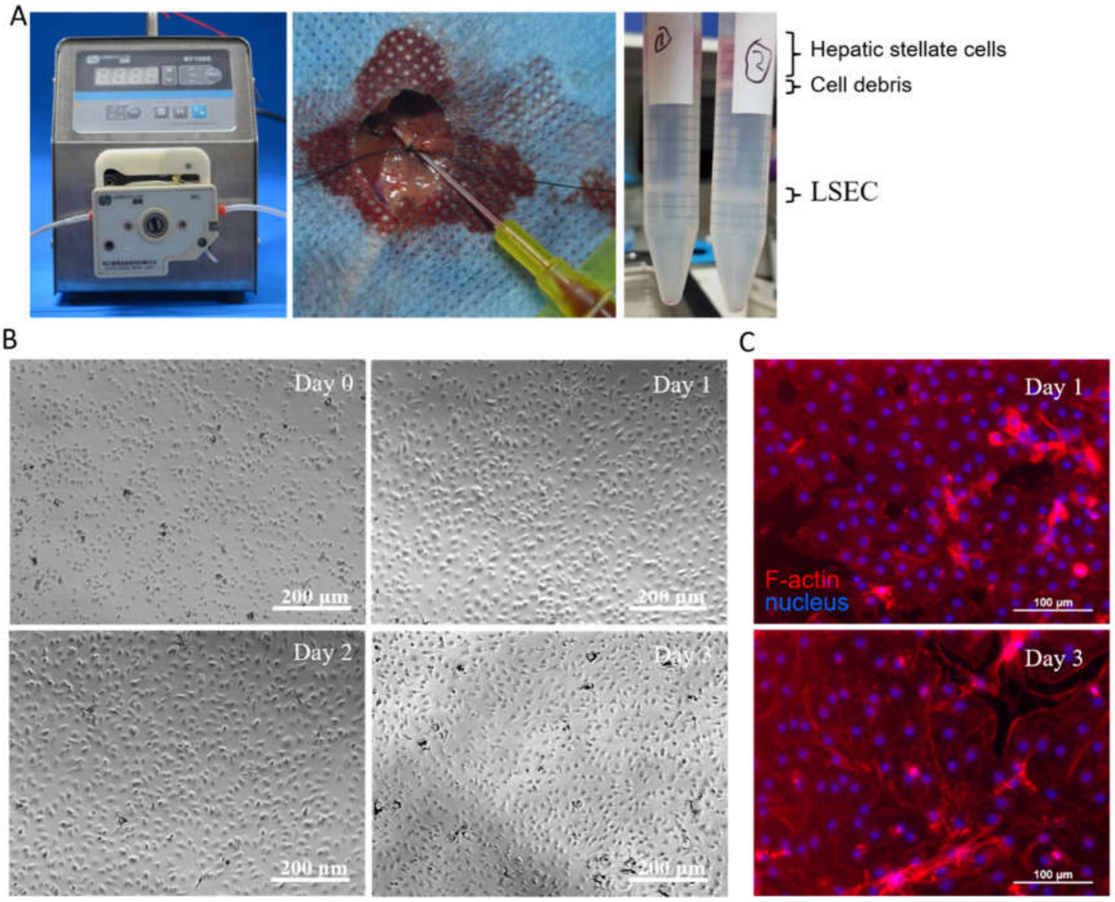

Supplementary Figure 1. Isolation and in vitro culture of mouse primary liver sinusoidal endothelial cells (p-LSECs) (A), Representative of images showing isolating p-LSECs by using in situ perfusion methods. **(B)**, Representative bright field images of p-LSECs cultured on plastic cell culture plates. Scale bars, 200 µm. **(C)**, Representative F-acting staining images of p-LSECs cultured on plastic cell culture plates. Scale bars, 100 µm.

Methods

Animal experiments.

C57BL/6 mice were purchased from the Laboratory Animal Resources Center at Tsinghua University and housed in the specific-pathogen free animal facility. The mice were maintained on a 12/12-hour light/dark cycle, 22-26 °C with sterile pellet food and water ad libitum. Institutional Animal Care and Use Committee (IACUC) of Tsinghua University approved all animal protocols used in this study. For mouse liver fibrosis model, fibrosis was induced by following a standard CCl_4 injection strategy with 4-week injection for early-stage fibrosis and 8-week injection for late-stage fibrosis.

Histological and Immunofluorescent staining

Paraffin-embedded human liver tissues were purchased from OriGene Technologies. Tissue sections were dried for 30 min at 60 °C, followed by deparaffinization and rehydration. Antigen retrieval was performed in sodium citrate buffer for 20 min in autoclave. Endogenous peroxidize activity was inhibited with 3% hydrogen peroxide. Tissue sections were permeabilized and blocked for 1 h followed by primary antibody incubation at 4 °C overnight. Biotinylated secondary antibodies were incubated at room temperature for 1 h and visualization was developed using

3,3′-diaminobenzidine. For immunofluorescent staining, fixed samples were permeabilized and blocked for 1 h followed by primary antibody incubation at 4 °C overnight. The used primary antibodies were listed as follows: anti-Transglutaminase-2 (Abcam, ab2386), anti-γ-GLU-ε-LYS (Enzo, CVL-MAB0012-Ps), anti-COL-1 (Abcam, ab6308), anti-Paxillin (Abcam, ab32084), anti-YAP (Abcam, ab52771). Filamentous actin (F-actin) was labelled using Acti-stain™ 488 phalloidin or Acti-stain™ 555 phalloidin (Cytoskeleton).

Quantification of TGM crosslinking degree

Mouse liver decellularized ECM were prepared by following an *in situ* perfusion protocol. Sodium dodecyl sulfate (SDS) solutions of concentration 0.01%, 0.05%, 0.1% were perfused into the liver tissue for about 36 h in total. The decellularized samples were washed by deionized water and were dried by CO_2 critical pointing drying strategy. The dried samples were sequentially digested by step-by-step enzymatic treatment. The following enzymes were used: collagenase I (GIBCO, 17100-017), pronase (Roche, 11459643001), papain (Sigma, P4762), aminopeptidase M (Shanghai Yuanye, S25360), prolidase (Sigma, P6675), aminopeptidase M, carboxypeptidase (Sigma, SAE0046). The digested samples were sedimented by cold methanol for 1 h and the pallets were discarded. The products in supernatants were collected and dried. Detection of γ-Glutamyl-ε-Lysine, hydroxyproline(HYP) was performed by HPLC-MS/MS. Quantification was performed by external standard methods. The crosslinking degree mediated by TGM is defined as the molar ratio (mole / mole) of γ-Glutamyl-ε-Lysine to the tropocollagen molecules detected in the sample.

Construction of the crosslinked collagen matrix.

All the reagents were pre-cooled by ice bath for about 1 h. For preparing un-crosslinked collagen matrix (UC), the type I collagen (Rat tail, Corning) was diluted to a final concentration of 2 $mg \cdot ml^{-1}$ and then was neutralized and adjusted to pH 7.4. The solutions were gently mixed by pipette in ice bath and then were transferred into customized molds. Gelation was performed by incubating the pre-mixed solutions at 37 °C for 3 hours in a humidified atmosphere. Additional PBS was gently added to the surface of collagen gel. For preparing TGM-crosslinked collagen matrix (TC), additional transglutaminase (Sigma-T5398; BomeiBio) was supplemented into the pre-mixed solutions of final enzymatic activity higher than 0.2 U ml^{-1}. The quality control should be performed by AQMC assay to confirm to crosslinking degree as indicated by Figure 1H. To prepare TGM-crosslinked collagen matrix with additional inhibition of crosslinking (TC^{in}), additional cystamine (Macklin) to was supplemented into the pre-mixed solutions during preparation. All the processes should be operated in ice baths.

Collagenase-mediated degradation assays.

Collagenase type I solutions of 2 $mg \cdot ml^{-1}$ were pre-warmed to 37°C in a thermostatic shaker. Prepared collagen matrix samples were placed in filter molds with mesh size of 30 μm. The filter molds were immersed in collagenase solutions and then being slowly shaken in the thermostatic shaker at 37°C. The part of collagen matrix having been degraded were determined by protein released out of the filter molds, which was quantified by BSA kit.

Optical Tweezer Measurements. Viscoelasticity of the collagen matrix was characterized by mmi CellManipulator optical trapping system (mmi Cellmanipulator, MM1 AG, Zurich, Switzerland). Uncoated polystyrene beads (d = 5 μm, hugebio) were embedded in collagen matrix during the preparation and were manipulated by an optical trap. The laser beam (8 W, 1,070 nm) was tightly focused through a series of Keplerian beam expanders and a high-N.A. objective (60 × 1.2; water; Nikon). A high-resolution quadrant detector was used for position detection. Calibration of the linear region of the detector and the trap relative elastic modulus (60 $pN \cdot nm^{-1}$) was performed in PBS using the same type of beads according to the harmonic oscillation method. After a bead being trapped by the laser, the

trapping laser was moved along a certain direction at a constant velocity of 1 $\mu m \cdot s^{-1}$ to manipulate the bead. The force-displacement curve was recorded. The matrix stiffness of the collagen matrix was determined by the slope of the linear region of force-displacement curve. The matrix stiffness was linearly correlated with the young's modulus of the collagen matrix. To determine the stress relaxation rate of collagen matrix, the bead was manipulated by an optical trap as described above until the detected force reached 30 pN. Then the bead was kept static while the load was recorded as a function of time. The stress relaxation data of the collagen matrix were fit to a two-element Maxwell–Weichert linear viscoelastic model with 2 Maxwell elements in parallel. The time for the initial stress to be relaxed to half its value during a stress relaxation test was defined as half stress relaxation time ($\tau^{1/2}$).

RNA isolation and real-time qPCR analysis. Total RNA extraction reagent (Vazyme) was used to isolate the RNA was isolated by using a total-RNA extraction kit (Vazyme). For samples of cell-embedded collagen matrix, samples were treated by total RNA extraction reagent with additional vortex for around 20 min. cDNA samples were prepared using a Hiscript II qRT SuperMix Kit (V) (Vazyme). Real-time qPCR experiments were performed using AceQ qPCR SYBR green master mix (Vazyme) in a CFX96 Real-Time PCR Detection System (Bio-Rad). The primer used are listed as follows:

Gene	Forward primer	Reverse primer
H-GAPDH	GAAGGTGAAGGTCGGAGTC	AAGATGGTGATGGGATTTC
H-DDR2	GATGTATGCCTACCACCGGG	ATGGAGGTTTCATCCAGGGG
H-PDGFA	GCAAGACCAGGACGGTCATTT	GGCACTTGACACTGCTCGT
H-COL3A1	GGAGCTGGCTACTTCTCGC	GGGAACATCCTCCTTCAACAG
m-18s rRNA	AGAAACGGCTACCACATCCAA	GGGTCGGGAGTGGGTAATTT
m-Ddr1	TGGTGGGCCTGGATGATTTC	GTGGGGAAGCTCTGATTGCT
m-Ddr2	ACCCACCACCTATGATCCCA	GCCACCAAGCAACCAATCAG
m-Trem2	CTGGAACCGTCACCATCACTC	CGAAACTCGATGACTCCTCGG
m-Ccr2	ATCCACGGCATACTATCAACATC	TCGTAGTCATACGGTGTGGTG
m-Mmp13	TGATGATGAAACCTGGACAAGCA	GGTCCTTGGAGTGATCCAGACCTA

Isolation of primary mouse liver sinusoidal endothelial cells.

Primary mouse liver sinusoidal endothelial cells were isolated 8-week-old mice using a modified in situ perfusion protocol. Briefly, cell suspension could be obtained after serial in situ and ex vivo digestion by pronase and collagenase I. The hepatocytes were eliminated through centrifugation at 50 g for 5 min twice. The supernatant containing non-parenchymal cells was then centrifuged at 580 g for 10 min at 4 °C. The cell pellet was re-suspended in 1640 medium and centrifuged at 1,200 g for 20 min through a 25%/50% percoll gradient (SolarBio). The sinusoidal endothelial cells were located at the interface at the 25%/50% percoll gradient. The cell fraction was isolated gently and was placed in an acid-washed glass Petri dish and incubated at 37 ◦C for 15 min. The non-adherent were then harvested and transferred to culture dishes or seeded onto collagen matrix. The morphology of isolated primary mouse liver sinusoidal endothelial cells was characterized by F-actin staining as indicated by supplementary figure 1.

Mouse Bone-Marrow-Derived Macrophages (BMDM) isolation.

Mouse BMDM were prepared from healthy male mice (8–12 weeks old C57BL/6). Bone marrow from femurs and tibiae was collected and filtered through a 40 μm cell strainer to remove debris. The cells were resuspended in DMEM medium containing 10% heat inactivated FBS, 1% Penicillin–Streptomycin solution (Gibco), and 15% L929-conditioned medium and distributed into 100 mm Petri-dishes. The cells were cultured for 7 days and yielded a

macrophage ($CD11b^+$ $F4/80^+$) population up to 95% as assessed by flow cytometry.

Statistics. Statistical analyses were performed using GraphPad Prism (version 8.4.2). Data are presented as the mean with standard deviation (s.d.). Statistical significance was determined using a two-tailed unpaired Student's t-test for the comparison of two groups or by ANOVA for comparison between multiple groups. Exact P values are marked in the figures.
